# Dataset for vulnerability model analysis in economically depressed areas

**DOI:** 10.1016/j.dib.2022.108307

**Published:** 2022-05-23

**Authors:** Zambrano-Yépez Claudia, Guillén-Rodríguez Yaritza

**Affiliations:** Universidad Laica Eloy Alfaro de Manabí, Manta, Ecuador

**Keywords:** Economically depressed areas, Vulnerable sectors, Socioeconomic characterization, Unemployment, Underemployment, Unsatisfied basic needs

## Abstract

This paper presents data for the application of the vulnerability model in economically depressed areas, through social, economic and capacity variables, with the objective of promoting public policies that contribute to the economic development of the territory and its integration into the labor market [1]. We used the family and housing databases of the Socioeconomic and Environmental Characterization Survey of the community of San Juan in the city of Manta [2]. The study population comprises the inhabitants of Sitio San Juan in the city of Manta, due to the conditions of exclusion due to the negative evaluations of the place (oxidation ponds, municipal slaughterhouse, garbage dump, coal processing, among others), and due to "the scarce existence of databases of vulnerable sectors in the province of Manabí" [3], considering that the last national census was conducted in 2010. As for the sample design, the census-type sweep technique was applied. The data set helps to determine the vulnerability conditions of the territory, for the application of the proposed model; in addition, these data can be used to analyze the determinants of the conditions of unemployment, underemployment and poverty in which the inhabitants of the sector live. Likewise, they can be complemented with future research in regions with similar characteristics in Ecuador or other territories to promote public policies that allow them to improve their living conditions and encourage public or private investment for the generation of employment and poverty reduction.

## Specifications Table

**Table:** 

Subject	Economics, Econometrics and Finance
Specific subject area	Economic Development and Growth
Type of data	Table Excel file Do-files for the Stata code
How data were acquired	Data were obtained from the survey based on two questionnaires (housing and family) prepared by a group of researchers.
Data format	Raw Analyzed
Parameters for data collection	Social, economic, and capacity variables were considered to determine the vulnerability conditions of the territory.
Description of data collection	Five complementary files are presented: the first contains the socioeconomic characteristics of the head of household and the population; the second analyzes the indicators of the socioeconomic vulnerability model; the third compiles the data in Excel for export to Stata; the fourth shares the do-file for the Stata code; and the fifth contains the original questionnaires applied in the survey. Table 1 contains the description of the variables extracted for the vulnerability model. Table 2, the socioeconomic characteristics of the head of household. Table 3, the vulnerability model. Table 4, household housing characteristics. Table 5, overcrowding per dwelling. Table 6, the availability of basic services in the dwellings. Table 7, the occupation of the head of household. Table 8, the average unemployment rate in the household. Table 9, the unemployment and underemployment rate of the population. Table 10, the educational level of the population and Table 11, the description of the indicators of the vulnerability model.
Data source location	Institution: Universidad Laica Eloy Alfaro de Manabí City/Town/Region: Manta / San Juan / Manabí Country: Ecuador Primary data sources:https://carreras.uleam.edu.ec/economia/encuesta-de-caracterizacion-socioeconomica-y-ambiental-del-sitio-san-juan-de-la-ciudad-de-manta-encarsoeca-2018/
Data accessibility	With the article: Excel file: Complementary 1: Socio-economic criteria Excel fie: Complementary 2: Unsatisfied basic needs Excel fie: Complementary 3: Document imported to Stata Do-files for the Stata code: Complementary 4 PDF file: Complementary 5: Original Questionnaires (translated) The raw data are deposited in: Repository name: Mendeley Data Data identification number: https://doi.org/10.17632/wbmy9227zh.3 Direct URL to data: https://data.mendeley.com/datasets/wbmy9227zh/3

## Value of the Data

• The data analyzed allow us to understand the persistent vulnerability in economically depressed sectors, as well as the rates of unemployment, underemployment, and unsatisfied basic needs, which measure the variables, social, economic, and household capacities of the San Juan de Manta community.
• The data could motivate the insertion of projects of various actors of society (academy, public entities, NGOs, among others) to the economic reactivation of the sector; in addition, they are important for the competent entities to formulate public policies that meet the needs of vulnerable sectors, thus promoting the development of their territories.
• The data set could encourage the creation of deeper lines of research on the relationship among the determinants of employment, underemployment, and unemployment.
• The data motivate the investigation of the conditions that characterize economically depressed areas, the application of models that allow to know the socioeconomic vulnerability of the territories, as well as the potential of the production capacities of their inhabitants, in such a way that they contribute to the economy of their territories.
• For [4], inequalities in the labor market and income distribution, as well as gender, ethnic and racial inequalities, lead workers to present vulnerabilities that increase poverty levels, especially in underprivileged territories, where the population tends to be deprived of access to basic services or is at greater risk of being deprived of such services, exposing them to the so-called "poverty and inequality traps" [5]. The importance of implementing the vulnerability model allows us to know the uncertainty of the poor population and the risk that the non-poor population presents of falling into poverty [6]. In addition, this model focuses on territorial spaces that have been segregated from the rest of the city and that live in precarious conditions, due to the environmental characteristics of the sector and the economic activities they develop.

## 1 Data Description

The data were extracted from the "San Juan Family Database" and the "San Juan Housing Database". The data set is presented in five complementary files: the first one compiles the variables: neighborhood, family composition, age, type of relationship, educational level, occupation of the head of household, work activity (working, not working), self-employment and source of income; variables to be processed: gender, age of the head of the household, educational level of the head of the household, educational level of the population group, economically active population, unemployment rate, underemployment rate, total household income. The second file collects the variables: Development Bonus, population identified as "Montubio", general IESS, educational level, branches of activity (primary and secondary sector), type of construction, floor material, rooms, family one, family two, family three, family four, latrines, well, river, tanker, rainwater, and persons per family; thus, the indicators of the socio-economic vulnerability model are processed. The third supplementary file presents the dataset exported to Stata for the social and economic characterization, as well as the unemployment and underemployment rate of the population; the fourth supplementary file presents the do-file for the Stata code. The fifth supplementary file presents the original questionnaires translated into English. Table 1 presents the description of the variables extracted and the data source for the construction of the vulnerability model.

**Table 1:** Description of the variables extracted for the vulnerability model.

Variables	Data Type	Description	Data Source	Data File Location
Development Bonus	Numeric	Number of people receiving the Human Development Bonus; obtained by replacing the value of $50 (equivalent to the amount of the BDH received by one person) by 1.	San Juan Family Database	Complementary File 2-Sheet 4 (Beneficiaries of the Human Development Bond)
Montubio population	Numeric	Number of people identified as Montubio	San Juan Family Database	Complementary File 2- Sheet 4 (Social Group identified as montubio)
IESS Affiliates	Numeric	Number of people IESS Affiliates	San Juan Family Database	Complementary File 2- Sheet 4 (IESS Affiliates)
Economically Active Population	Numeric	Number of people of working age (population over 15 years old and under 65 years old)	San Juan Family Database	Complementary File 2- Sheet 4 (Economically Active Population)
Population with Higher Education	Categorical	Number of people with higher and post-graduate education. It is obtained from Categories 6 and 7 of the educational level of the questionnaire.	San Juan Family Database	Complementary File 2- Sheet 4 (from columns F to Y)
Branches of activity	Numeric	Number of persons employed in activity branch 1, activity branch 2, activity branch 3, activity branch 4 and activity branch 5, corresponding to the activities of the first and second sectors of the economy.	San Juan Family Database	Complementary File 2 - Sheet 4 (from column Z to AD)
Type Construction	Categorical	Number of households with reed or reed-covered walls. Obtained from category 1 and 4 of the construction type in the questionnaire.	San Juan Housing Database	Complementary File 2 - Sheet 5 (Construction type)
Material Floor	Categorical	Number of households with ground, cane or plank. It is obtained from categories 4, 5 and 6 of the housing floor material in the questionnaire.	San Juan Housing Database	Complementary File 2 - Sheet 5 (Floor Material)
Rooms	Numeric	Number of rooms in the households	San Juan Housing Database	Complementary File 2 - Sheet 5 (Bedrooms)
Inhabitants in the home	Numeric	Number of family members living in the home	San Juan Housing Database	Complementary File 2 - Sheet 5 (from column E to H)
Persons per Room	Numeric	Total inhabitants in the household/rooms (Calculated variable).	San Juan Housing Database	Complementary File 2 - Sheet 5 (Household members per bedroom)
Access to Basic Services	Numeric	Number of households with latrines, and where water comes from wells, rivers, tanks or rainwater. It is obtained from category 6 in housing infrastructure and from category 2,3,4 and 5 in the origin of the water in the questionnaire.	San Juan Housing Database	Complementary File 2 - Sheet 5 (from column L to P)
Households with deficient basic services	Numeric	Number of households with deficient basic services (Calculated variable)	San Juan Housing Database	Complementary File 2 - Sheet 5 (Households with deficient Basic Services)
People per family	Numeric	Number of persons per family	San Juan Family Database	Complementary File 2 - Sheet 5 (People per Family)

The socioeconomic characteristics of the head of household are presented in Table 2, with the variables: Neighborhood, age, gender, marital status, and educational level. In the supplementary file 1 (Sheet 4) the data analyzed in Table 2 are available and in sheet 3 the data extracted from the aforementioned database.

**Table 2:** Socioeconomic characteristics of the head of household.

Features	Freq.	Percent
Neighborhood		
San José	71	11,47%
San Juan	295	47,66%
San Ramón	55	8,89%
Santa Marianita	161	26,01%
Valle Claro	37	5,98%
Age of Head of Household		
15 to 25 years	55	8,89%
26 to 35 years	136	21,97%
36 to 45 years	153	24,72%
46 to 55 years	110	17,79%
56 to 65 years	66	10,66%
66 years and over	99	15,99%
Gender		
Female	94	15,19%
Male	525	84,81%
Marital Status		
Married	346	56,54%
Common-law marriage	144	23,53%
De facto union	3	0,49%
Widower	42	6,86%
Separate	41	6,70%
Divorced	14	2,29%
Single	22	3,59%
S/D	7	1,13%
Education Level		
Incomplete primary	175	28,27%
Complete primary	241	38,93%
Incomplete Secondary	56	9,05%
Full secondary	87	14,05%
Higher	10	1,62%
Illiterate	49	7,92%
S/D	1	0,16%

Table 3 presents the data analyzed from the survey, to determine the indicators of the vulnerability model for economically depressed areas (Supplementary file 2). It consists of social, economic and capacity variables, which measure poverty by unsatisfied basic needs (including housing characteristics, overcrowding and accessibility to basic services); the population that receives economic assistance from the Government, with the Development Bonus; the population that identifies itself as part of the "Montubio" ethnic group^1^; proportion of primary and secondary sector activities^2^; informality, which is measured by the number of people affiliated with the Ecuadorian Social Security Institute (IESS) in relation to the Economically Active Population (EAP). For the EAP, persons over 15 years of age and under 65 years of age are considered (Supplementary file 1 - Sheet 6); finally, the educational levels of family members are used, and only those with higher and postgraduate education are considered. The data to calculate the weighting of the variables are available in the supplementary file 2 (sheets 4 and 5).

**Table 3:** Vulnerability model for depressed economic zones.

Social Variables		Consensual Weighting
% Poverty due to Unsatisfied Basic Needs	(+)	0,80
Number of poor by Unsatisfied Basic Needs	(+)	0,85
% Human Development Bonus Beneficiaries	(+)	0,02
Border Cantons		Does not apply
% Montubia Population	(+)	0,02

Economic Variables		Consensual Weighting

Primary Activity/Economically Active Population	(+)	0,07
Secondary Activity/Economically Active Population	(+)	0,28
Informality (Affiliates/ Economically Active Population)	(+)	0,26
Credit by canton		Does not apply

Capacity Variables		Consensual Weighting

Economically Active Population	(+)	0,66
Human Capital	(+)	0,03

The Vulnerability Model states that the following variables are considered for the calculation of poverty due to unsatisfied basic needs: housing, overcrowding and accessibility to basic services. Table 4 shows the characteristics of the houses: predominant material of the walls, for the vulnerability model, only those made of reed and coated reed are considered, which show the level of poverty; as for the predominant floor of the houses, those made of soil, reed and wood are considered. The data show that 95 houses meet these characteristics. It should be noted that Table 4 totals 120 dwellings since some households meet all the characteristics of both the floor and the walls. The data can be found in Supplementary file 2 (sheet 5).

**Table 4:** Housing characteristics.

	Freq	Percent
Housing Material		
Cane	44	8,40%
Concrete	58	11,07%
Brick or block	347	66,22%
Coated reed or wood	17	3,24%
Pure Wood	26	4,96%
Mixed	26	4,96%
Other material	6	1,15%
Housing floor		
Duela/parquet/plant	4	0,76%
Ceramics/tile	135	25,76%
Brick cement	326	62,21%
Board	34	6,49%
Cane	13	2,48%
Earth	12	2,29%

Table 5 describes the overcrowding data, obtained from the total number of household members in relation to the number of rooms in the dwelling, considering that [9] establishes that the household is overcrowded when the number of people per bedroom is greater than three.

**Table 5:** Housing overcrowding.

Overcrowding by dwelling	Freq	Percent
0 to 3 Household members per bedroom	454	86,64%
More than 3 Household members per bedroom	70	13,36%

Table 6 describes the accessibility of basic services, through the sanitary conditions of the house. For the analysis of these data, we consider the dwellings that have latrines (or do not have sanitary facilities), as well as the origin of the water received by the dwelling (well, river, tank, rainwater, and any other source other than the public network). Supplementary file 2 (Sheet 5) shows the data presented in Table 6.

**Table 6:** Accessibility to basic services.

Availability of basic services	Freq	Percent
Have basic services	268	51,15%
Do not have basic services	256	48,85%

Table 7 presents the occupational activities of the head of the household, highlighting unskilled workers; and service workers and traders. From the data set, the 12 occupations are considered, categorized from 1 to 12 and are included in supplementary file 1 (Sheet 4).

**Table 7:** Occupation of the head of household.

Occupation of the Head of the Family		
Management staff of the Public Administration and companies	3	0,48%
Scientific and intellectual professionals	1	0,16%
Medium level technicians and professionals	8	1,29%
Office clerks	5	0,81%
Service worker and merchants	120	19,39%
Skilled agricultural and fishing worker	12	1,94%
Operators and artisans’ officers	14	2,26%
Plant and machine operators	13	2,10%
Unskilled workers	351	56,70%
Unemployed	45	7,27%
Inactive	47	7,59%

Table 8 shows the ranges of the average unemployment rate per household and the total economic income each household receives. The average household unemployment rate is obtained from the number of persons not working in relation to the EAP per household. For total income, all income items received by households are considered (wages and salaries, informal work - agriculture, informal work - non-agriculture, other jobs, Development Bonus, Joaquín Gallegos Lara Bonus,^3^ in-country shipments, and gifts). These data are available in Supplementary file 1 (Sheet 7). It is important to note that, according to [10], for the year 2021, the basic family basket for a typical household of four people is 709.40 USD; and the vital family basket is 499.89 USD.

**Table 8:** Average unemployment rate per household and total family income.

Average unemployment rate at home		
0 to 25%	420	68,17%
26 to 50%	166	26,66%
51 to 75%	22	3,55%
76 to 100%	11	1,62%
Total income		
0 to 400 USD	434	70,11%
401 to 800 USD	151	24,39%
801 to 1200 USD	25	4,04%
1201 to 1600 USD	6	0,97%
1601 to 2000 USD	2	0,32
3601 to 4100 USD	1	0,16%

Table 9 presents the average unemployment rate of the population; the data on non-working persons per household are considered in relation to the working-age population; and, for the underemployment rate, data on self-employed persons in relation to the number of working persons is used; in both cases, the average is calculated using Stata software. The data can be found in Supplementary file 1 (Sheet 6).

**Table 9:** Unemployment and underemployment rates in the population.

Variables	Agreed Weighting
Average unemployment rate in the population	0,17
Average rate of underemployment in the population	0,56

Table 10 shows the educational levels of the total population (the description of this variable can be found in Supplementary file 1 - Sheet 1); the data are grouped using formulas in Microsoft Excel, which are available in Supplementary file 1 (Sheet 5).

**Table 10:** Educational level of the population.

Family	NEL (−3 years)	Illiterate	Elementary School incomplete	Elementary School completed	High School incomplete	High School completed	Superior	Postgraduate	No answer	TOTAL
Husband		41	138	209	44	78	10		1	521
Wife		29	138	258	60	79	12		1	577
Sons	77	3	174	87	107	94	16			558
Daughters	66	3	155	47	94	56	23	2		446
Other family	15	8	44	16	14	14	3		1	115
Total	**158**	**84**	**649**	**617**	**319**	**321**	**64**	**2**	**3**	**2.217**
Percentage	**7,1**	**3,8**	**29,3**	**27,8**	**14,4**	**14,5**	**2,9**	0,1	0,1	100
Accumulated percentage	**7,1**	**10,9**	**40,2**	**68,0**	**82,4**	**96,9**	**99,8**	99,9	100,0	

## 2 Experimental Design, Materials and Methods

### 2.1 Description of the Study Area

The city of Manta is the second most populated city in the province of Manabí, characterized for being an important seaport located on the central-south coast of Ecuador, facing the Pacific Ocean, in the center of the coastal region, which presents an accelerated demographic growth due to the dynamics of its economy developed by the fishing industry, vegetable oils, assembly plants and tourism. San Juan community is located southeast of the city and is part of its urban zone, where the municipal slaughterhouse, the garbage dump, 12 oxidation ponds, and several recycling companies are located, contributing to the environmental contamination of the sector and the city, due to bad odors and the proliferation of insects, which is aggravated by the inhabitants' activities of raising animals (chickens, pigs, and goats) and making charcoal without any restrictions or controls.

Approximately 2217 people live in San Juan community, distributed in five neighborhoods: Santa Marianita, San Juan, San José, San Ramón and Valle Claro; due to the previously described conditions that characterize the place, in the opinion of [11] "suffer multiple processes of discrimination: social class, ethnicity, and even environmental discrimination (topographically marginalized territories)" (p. 21) and consequently "become communities of recyclers", where informality, unemployment and underemployment predominate.

### 2.2 Collection of the Data Set

For the application of the survey, the census-type sweep technique was used in the five neighborhoods that make up the San Juan site. The survey was applied on February 1, 2019 from 08H00 to 16H00. The instrument used contains three parts: housing characteristics, environmental problems and household data. It collects a total of 71 questions of closed, open and multiple choice type, based on the Household Surveys and Population and Housing Census forms of the National Institute of Statistics and Census of Ecuador. For its application we counted with the participation of research professors of Economic Sciences, Agroindustry and Psychology of the Universidad Laica "Eloy Alfaro" of Manabí, 207 student surveyors, 19 student researchers in training, leaders of each neighborhood who were in charge of socializing and coordinating actions with the inhabitants of the sector and helped in the logistics and coordination in the collection of information. The surveys were conducted to the total population of the San Juan site, obtaining a population of 2217 inhabitants in 619 households.

### 2.3 Socioeconomic Vulnerability Model

The construction of the vulnerability model is based on the methodology developed by [1], which includes social, economic and capacity variables. The first includes four indicators: percentage of poverty by Unsatisfied Basic Needs, number of poor by Unsatisfied Basic Needs, percentage of receptors of the Human Development Bonus and percentage of montubia population; the second considers two indicators: Percentage of the economically active population in the primary and secondary sectors; finally, the Capabilities variable considers two indicators: percentage of the economically active population and percentage of the population with higher education. Table 11 reveals the procedure used to obtain each indicator.

**Table 11:** Description of the indicators of the vulnerability model.

Social Variables	Indicators	Procedure
Poverty	% Povertyby UBN	Divide the number of households with unsatisfied basic needs by the total number of households.
	% Number of poor by UBN	Divide the population with unsatisfied basic needs by the total number of residents.
Human Development Bonus Beneficiary	% of beneficiary of the Human Development Bonus	Data is extracted from the Development Bonus variable.
Priority population	% of the montubia population	Divide the number of people identified as Montubio by the total number of residents.

Economic Variables	Indicators	Procedure

Economic Structure	% of population in the Primary Sector	Divide the sum of persons engaged in labor activities belonging to the first and second branches of economic activity by the Economically Active Population.
	% of population in the Secondary Sector	Divide the sum of persons engaged in labor activities belonging to the second, third and fourth branches of economic activity by the Economically Active Population.
Informality	% of informality	Divide the number of IESS members by the Economically Active Population.

Capacity Variables	Indicators	Procedure

EAP	% of Economically Active Population	Divide the Economically Active Population by the total number of residents.
Human Capital	% of population with higher education	Divide the sum of people with higher education and postgraduate studies by the total population.

The social variables include: the percentage of poverty due to unsatisfied basic needs and the number of poor due to unsatisfied basic needs, obtained from households with deficient housing characteristics, overcrowded homes, and accessibility to deficient basic services; percentage of beneficiaries of the Development Bonus and Montubia population. In the analysis of economic variables, to determine the economic structure of the territory, the proportion of the population engaged in activities in the primary and secondary sector is considered; and considering social security (Affiliated to the IESS) informality is determined, denoting the unemployment or underemployment of the locality. The capacity variables are determined based on the proportion of the economically active population and the human capital index, which is complemented by the presence of productive or non-productive activities in the territory.

## Ethics Statement

The raw data in this article come from open data sources, thus fulfilling the ethical requirement to be published in Data in Brief magazine.

## CRediT authorship contribution statement

**Zambrano-Yépez Claudia:** Conceptualization, Methodology, Writing – original draft. **Guillén-Rodríguez Yaritza:** Software, Data curation, Validation.

## Declaration of Competing Interest

The authors declare that they have no known competing financial interests or personal relationships which have or could be perceived to have influenced the work reported in this article.

## Data Availability

Encuesta de caracterización socioeconómica y del Sitio San Juan de la ciudad ambiental de Manta (Original data) (Mendeley Data) and Encuesta de caracterización socioeconómica y del Sitio San Juan de la ciudad ambiental de Manta (Reference data) (FAIRsharing.org).

## Appendix: Supplementary Materials

Complementary 1

Complementary 2

Complementary 3

Complementary 4

Complementary 5

## Appendix B Supplementary materials

Image, application 1

Image, application 2

Image, application 3

Image, application 4

Image, application 5

## Footnotes

1 In Ecuador, according to Article 59 of the Constitution [7], the rights of the Montubios are recognized with the objective of preserving their culture and identity; by virtue of this, the Montubio people of Ecuador receive constitutional recognition [8].

2 Agriculture and mining: primary sector; manufacturing industries, water distribution, and construction: secondary sector.

3 Granted to family members of persons with very severe disabilities.
