# Supplementary material for: Dataset for vulnerability model analysis in economically depressed areas
Source: Data Brief. 2022 May 23;42:108307. doi: 10.1016/j.dib.2022.108307 (PMC9160495; doi:10.1016/j.dib.2022.108307)
Supplement: Supplementary file 5 [file mmc5.pdf]

UNIVERSIDAD LAICA ELOY ALFARO DE MANABÍ

Research Commission
Commission of Linkage with the Society

Economics career
Faculty of Agricultural Sciences
Faculty of Industrial Engineering
Faculty of Nursing
Language Therapy Career

Project: Socioeconomic characterization of the San Juan site in the city of Manta.

Questionnaire for Socioeconomic and Environmental Survey

OBJECTIVE:
Identify socioeconomic and environmental needs in vulnerable sectors to facilitate the orientation of future strategies aimed at improving the quality of life and community development.

GENERAL DATA

Name and last name: ____________________________ ____________ID number:____________________

Neighborhood: ______________________________________ ________Telephone:____________________

Relationship with the Head of the Household: ____ __________

FAMILY CODE

FAMILY COMPOSITION

1. How many people does your family consist of? (   )

2. How the family is conformed? **Specify the number**
   - 2.1. Spouses Sex M( ) F ( )
   - 2.2. Children Sex M( ) F ( )
   - 2.3. Grandchildren Sex M( ) F ( )
   - 2.4. Son-in-law Sex M( ) F ( )
   - 2.5. Daughter-in-law Sex M( ) F ( )
   - 2.6. No housing Sex M( ) F ( )
   - 2.7. Domestic employee Sex M( ) F ( )
   - 2.8. Other relative Sex M( ) F ( )
   - 2.9. Grandparents Sex M( ) F ( )
   - 2.10. Other non-relative Sex M( ) F ( )
3. Of the family members, to which ethnic group do they belong?
   - 3.1. Indigenous (   )
   - 3.2. Afro-Ecuadorian (   )
   - 3.3. Montubio (   )
   - 3.4. Mixed race (   )
   - 3.5. White (   )
   - 3.99. Others (   )
4. What is the age of the family members? **Write the age of each family member**

MEMBERS	
4.1.Husband	
4.2.Wife	
4.3.Sons	1st__2nd_3rd__<br>4th__5th_6th____
4.4.Daughters	1st__2nd_3rd__<br>4th__5th_6th____
4.5.Others	1st__2nd_3rd__<br>4th__5th_6th____

5. The characterization by age groups of the family behaves as follows. **Identify the number by groups**

	AGE	
**5.1.**	0 to 1	
**5.2.**	1 to 5	
**5.3.**	5 to 12	
**5.4.**	12 to 19	
**5.5.**	19 to 40	
**5.6.**	40 to 65	
**5.7.**	65 and over	

6. What religion do the family members profess? **Write the number of members in each option.**

**RELIGION**	
6.1.Catholic	
6.2.Evangelical	
6.3.Jehovah'sWitness	
6.4.Mormon	
6.5.Atheist	
6.6.Other	

7. How often do you attend religious services?

**Frequency**	
7.1.Weekly	
7.2.Fortnightly	
7.3.Monthly	
7.4.Occasionally	
7.5.Commitments	
7.6.Never	

8. What is the level of education of the family members who have had access to any educational system? **Write the number of people in each option.**

	**(- 3 years)**	**Incomplete elementary school**	**Primary complete**	**Secondary incomplete**	**Secondary complete**	**High School**	**Post-graduate**	**Illiterate**
8.1.Husband								
8.2.Wife								
8.3.Sons								
8.4.Daughters								
8.5.Others								

9. What is the number of family members who are Digital Illiterate?
   9.1. Number of Digital Alphabets (Education) per family ( )

10. What is the type of relationship of the couples?
   10.1. Married ( )
   10.2. Free Union ( )
   10.3. Common-law relationship ( )
   10.4. Widowed ( )
   10.5. Separated ( )
   10.6. Divorced ( )
   10.7. Single ( )
   10.8. Other (specify) ____

11. What are the socioeconomic characteristics of the mothers of the families?
   11.1 Pregnant single mother ( ) Age ( ) Children ( ) Works ( ) Not working ( )
   11.2 Single mother ( ) Age ( ) Children ( ) Works ( ) Not working ( )

12. In total, how many **LIVE BORN** sons and daughters has the family had? ( )

13. Of all your sons and daughters, how many are **CURRENTLY ALIVE**? ( )

14. Number of deaths per family? ( )

15. Cause of neonatal death? Write the number.

   15.1. Asphyxia ____________
   15.2. Cardiorespiratory arrest ____________
   15.3. Congenital malformation____________
   15.4. doesn't know ____________
   15.99. Other **(Specify)** ____________

16. Cause of death of family members?
   - 16.1. Diabetes ____________
   - 16.2. Hypertension ____________
   - 16.3. Heart failure ____________
   - 16.4. Cancer ____________
   - 16.5. Traffic accident ____________
   - 16.6. Home accident ____________
   - 16.7. Occupational accident ____________
   - 16.8. Suicide ____________
   - 16.9. Cirrhosis ____________
   - 16.10. Not known ____________
   - 16.99. Other **(Specify)** ____________

17. What is the pathological history of the family?
   - 17.1. Diabetes ____________
   - 17.2. Hypertension ____________
   - 17.3. Heart failure ____________
   - 17.4. Respiratory insufficiency ____________
   - 17.5. Cancer ____________
   - 17.6. Cirrhosis ____________
   - 17.99.Other **(Specify)** ____________

18. What is the place of birth of the family members? Specify the number and place

**Member/Location**	**In this community**	**Province/Canton**		**Country**	
18.1. Husband					
18.2. Wife					
18.3. Sons					
18.4. Daughters					
18.5. Others					

19. How does the fundamental activity of the family members behave? Write in number of people per activity.

   - 19.1. Work ( )
     - 19.1.1.Full time ( )
     - 19.1.2. Haltime ( )
     - 19.1.2.Part time ( )
   - 19.2. Not working ( )
   - 19.3. Studying ( )
   - 19.4. Studying and working ( )
   - 19.5. Household executive ( ) Male ( ) Female ( )

20. What is the condition in which the work activity is performed? **Specify the number of persons.**
   - 20.1. Employer ( )
   - 20.2. Partner ( )
   - 20.3. Self – employed ( )
   - 20.4. Government/State Employee/Worker ( )
   - 20.5. Employee/Private Worker ( )
   - 20.6. Journeyman / Laborer ( )
   - 20.7. Craftsman ( )
   - 20.8. Unpaid household Worker ( )
   - 20.9. Unpaid Worker in Another Household ( )
   - 20.10. Unpaid salaried assistant / day laborer ( )

21. Indicate the work activity performed by working family members.
    21.1. Member1 ______________________
    21.2. Member2 ______________________
    21.3. Member3 ______________________
    21.4. Member4 ______________________
    21.5. Member5 ______________________
    21.6. Member6 ______________________

22. What is the branch in which you work? Specify the number of people **_DO NOT ANSWER_**
    22.1. Agriculture, forestry and fishing.. ( )
    22.2. Exploitation of mines and quarries...( )
    22.3. Manufacturing industries ....( )
    22.4. Supply of electricity, gas, steam and air conditioning..( )
    22.5. Water distribution; sewerage, waste management and sanitation activities.............( )
    22.6. Construction ....( )
    22.7. Wholesale and Retail trade; reappearance of motor vehicles and motorcycles...........( )
    22.8. Transportation and storage...( )
    22.9. Accommodation and food service activities..( )
    22.10. Information and communication..( )
    22.11. Financial and insurance activities..( )
    22.12. Real estate activities...( )
    22.13. Professional, scientific and technical activities..( )
    22.14. Administrative and support service activities..( )
    22.15. Public administration and defense; mandatory social security plans ....( )
    22.16.Teaching...( )
    22.17.Human health care and social work activities..( )
    22.18.Arts, entertainment and recreation...( )
    22.19.Other service activities..( )
    22.20.Household activities as employees ....( )
    22.21.Undifferentiated activities of households as producers of goods and services for own use.............. ( )
    22.22.Activities and organizations extraterritorial ....( )

23. Specify in number of people how many members of the family are working in activities related to:
    23.1. Recycling ( )
    23.2. Breeding and commercialization of animals ( )
    23.3. Agriculture ( )
    23.4. Trade and commerce ( )
    23.5. Own business (Indicate the activity)______________________
    23.99.Other (specify)___________________________________

24. How many of the family members are covered or enrolled in health insurance?
    24.1. General IESS ( )
    24.2. IESS volunteer ( )
    24.3. Farmer IESS ( )
    24.4. ISSFA Insurance ( )
    24.5. ISSPOL Insurance ( )
    24.6. Particular ( )
    24.99.Other (specify)__________________________

25. What type of affiliation or membership do family members have? Specify the name of the Organization
    25.1. Producers Association____
    25.2. Association of Recyclers____
    25.3. Association of traders____
    25.4. Club____
    25.5. Communal banks____
    25.6. Guild____
    25.7. Program____
    25.8. Women's Group____
    25.9. None____

26. What is the occupation of the Head of Household?

26.1. Management personnel of public administration and companies	
26.2. Scientific and intelectual professionals	
26.3. Technicians and mid-level professionals	
26.4. Office employees	
26.5. Service workers and traders	
26.6. Skilled agricultural and fishing worker	
26.7. Operative officers and craftsmen	
26.8. Plant and machine operators	
26.9. Unskilled workers	
26.10. Armed forces	
26.11. Unemployed	
26.12. Inactive Workers	

27. What is the amount of income, by source, received by household members? Specify in dollars per month

**Income**	
**27.1. CURRENT MONETARY INCOME**	
**Earned Income**	
27.1.1.Wages and salaries	
27.1.2.Agricultural self-employment	
27.1.3.Non-agricultural self-employment	
27.1.4.Income from other work	
**Property rental**	
27.1.5.House leases	
27.1.6.Lease of aparments	
27.1.7.Lease of land or other fixed assets	
27.1.8.Income from patents and copyrights	
**Income from capital**	
27.1.9.Interest on bonus	
27.1.10. Interest on savings accounts	
27.1.11. Interest on loans	
27.1.12. Interest on stock dividends	
**Transfers**	
27.1.13. Pensions for retirement, orphans, alimony, etc.	
27.1.14. Human Development Bonus	
27.1.15. Joaquin Gallegos Lara Bonus	
27.1.16. Money transfers from friends and relatives within the country	
27.1.17. Sending money from friends and family abroad	
27.1.18. scholarships	
27.1.19. Money provided by private or public institutions, NGOs or church	
**Other current revenues**	
**27.2. CURRENT NON-CASH INCOME**	
27.2.1.Wages in kind	
27.2.2.Self-consumption and Self-supply	
27.2.3.Gifts	
27.2.4.Imputed value of own and assigned housing	

28. How many family members migrated to another country? **Write the number of people**.

28.1.In the last 5 years ( )
28.2.6 years or more ( )

29. Of those who migrated, how many returned? ( )

30. What was the age of your family member when he/she left the country? Write the age for each person
    - 30.1. Age at leaving the country member 1 ( )
    - 30.2. Age at leaving the country member 2 ( )
    - 30.3 Age at leaving the country member 3 ( )
    - 30.4. Age at leaving the country member 4 ( )
    - 30.5 Age at leaving the country member 5 ( )
    - 30.6 Age at leaving the country member 6 ( )
31. What was the year of departure? Write the year of departure for each person
    - 31.1. Year of departure of member 1 ( )
    - 31.2. Year of departure of member 2 ( )
    - 31.3 Year of departure of member 3 ( )
    - 31.4. Year of departure of member 4 ( )
    - 31.5. Year of departure of member 5 ( )
    - 31.6. Year of departure of member 6 ( )

32. ¿What was the main reason for the trip?
    - 32.1.Work
    - 32.2.Study ( )
    - 32.3.Family union ( )
    - 32.4.Other (specify)______________________________

33. In which country are you currently living?
    - 33.1. __________________
    - 33.2. __________________
    - 33.3. __________________
    - 33.4. __________________
    - 33.5. __________________
    - 33.6. __________________

34.If you receive money from abroad, please indicate the following:
- 34.1.Amount____________________
- 34.2.Country____________________
- 34.3.Frequency__________________

35. Does any family member have any type of disability? **Write the number**

	Number	Has an ID card	%
35.1.Intellectual	( )	( )	( )
35.2.Physical - motor	( )	( )	( )
35.3.Visual	( )	( )	( )
35.4.Auditory	( )	( )	( )
35.5.Mental	( )	( )	( )

35.99. Other (specify)____

36. Does any member of the family suffer from a catastrophic illness? **Write the number of people**
    - 36.1. YES ( )
    - 36.2. NO ( )

37.If the answer is YES, what is the type of disease your family suffers from?_______ _______

38. What is the amount of money the family spends on the following expenses items? Specify in dollars per month
    - 38.1. Food and non-alcoholic beverages...( )
    - 38.2. Alcoholic beverages, cigarettes and narcotics...( )
    - 38.3. Clothing and footwear...( )
    - 38.4. Hosting, water, electricity, gas and other fuels ....( )
    - 38.5. Furniture, household items and home maintenance....................................( )
    - 38.6. Health ....( )
    - 38.7. Transportation...( )
    - 38.8. Communications..( )
    - 38.9. Recreation and Culture..( )
    - 38.10. Education..( )
    - 38.11. Restaurants and Hotels ....( )
    - 38.12. Miscellaneous and Services..( )
    - 38.99. Others____( )

39. Does any member of the family own a business?
    39.1. YES ( )
    39.2. NO ( )
40. If your answer is YES, please indicate if you have received any training on:
    40.1. Market research ( )
    40.2. Economy and/or finance ( )
    40.3. Cost accounting ( )
    40.4. Taxation ( )
    40.5. Quality Control ( )
    40.6. Customer service ( )
    40.7. Marketing ( )
    40.8. Has not received ( )
    40.99.Other________________________
41. Indicate the topics of interest to be a beneficiary of training projects
    41.1. Environmental ( )
    41.2. Productive ( )
    41.3. Business ( )
    41.4. Social ( )
    41.5. Educational - cultural ( )
    41.6. Economic ( )
    41.7. Tax accounting ( )
    41.8. Marketing ( )
    41.9. Business plans ( )
    41.10. Financial ( )
    41.11. Legal ( )
    41.12. Investment projects ( )
    41.13. Health ( )
    41.14. Constructive ( )
    41.99.Others____
42. From your point of view, what are the main problems affecting the San Juan de Manta Community?
    42.1. Social:____
    ____
    42.2. Economic:____
    ____
    42.3. Environmental:____
    ____
    42.4. Health: ____
    ____
    42.5. Educational - cultural ____ ______
    ____
    42.6. Business ____ _____
    ____
    42.99.Other ____ __________
    ____

On behalf of Uleam, Thank you.

WORK TEAM DATA: (Required information)

Name of the surveyor:_______________________________________Telephone:_______________

Career to which you belong:____

Name of the team supervisor:____

Career Coordinator:____

Date of data collection:____

CODE OF THE SURVEYEDHOUSE:____

______________________ ______________________

Supervisor signature (before) Supervisor Signature (after)

Project: Socioeconomic characterization of the San Juan site in the city of Manta

Questionnaire for Socioeconomic and Environmental Survey

OBJECTIVE:

Identify socioeconomic and environmental needs in vulnerable sectors to facilitate the orientation of future strategies aimed at improving the quality of life and community development.

GENERAL DATA

Name and last name: ____ID number:__________________

Neighborhood: ____ _______Telephone:__________________

Relationship with the Head of Household____ ___________

CODE

HOUSING DATA

1. What type of dwelling are you living?
   - 1.1. Own ( )
   - 1.2. Rented ( )
   - 1.3. Borrowed ( )
   - 1.4. Heritage ( )
   - 1.5. Family nucleus ( )
2. Is the home you are living in mortgaged?
   - 2.1. YES ( )
   - 2.2. NO ( )
   - 2.3. Don't know ( )
3. Do you have a deed for the land on which your home?
   - 3.1. YES ( )
   - 3.2. NO ( )
   - 3.3. Do not know ( )
   - 3.4. In legalization process ( )
4. What type of construction does the house have?
   - 4.1. Cane ( )
   - 4.2. Concrete ( )
   - 4.3. Brick or block ( )
   - 4.4. Coated cane or wood ( )
   - 4.5. Wood ( )
   - 4.6. Mixed ( )
   - 4.99.Others (specify)_______
5. What type of damage did your home suffer in the 16A earthquake?
   - 5.1. None ( )
   - 5.2. Moderate (fissurres) ( )
   - 5.3. Partial loss ( )
   - 5.4. Total loss ( )
6. Did you receive help to rebuild your house?
   - 6.1. YES ( )
   - 6.2. NO ( )
7. If the above answer is YES, indicate from which Institution or Organization
   - 7.1. Insurance (mortgaged house) ( )
   - 7.2. MIDUVI ( )
   - 7.3. Municipality (Relocated housing solution) ( )
   - 7.4. Foundation______________________________________ _____________________
   - 7.99.Other (specify)______________________________ ____________________________
8. What is the predominant FLOORING material in this house?
   - 8.1. Wood/parquet/plank/treated plank/floating floor ( )
   - 8.2. Ceramic/ tile/ vinyl Marble/ ( )
   - 8.3. Cement/brick ( )
   - 8.4. Untreated plank/plank ( )
   - 8.5. Cane ( )
   - 8.6. Earth ( )
   - 8.99. Other (specify) ____

9. What is the size of the land where your house is located, and what are the meters of construction?
   9.1. ______m2 of land
   9.2. _______m2 construction

10. Write the infrastructure number that corresponds to your home. Write the number in each option
    10.1. Floors ( )
    10.2. Bedrooms ( )
    10.3. Living room ( )
    10.4. Dining room ( )
    10.5. Bathrooms ( )
    10.6. Latrine ( )
    10.7. Shower ( )
    10.8. Garage ( )
    10.9. Yard ( )
    10.99Others (specify)____

11. What basic and complementary services does the house have? ***Rate from none=0 to optimum=5***

	0 1 2 3 4 5
11.1. Electricity	□□□□□□
11.2. Water	□□□□□□
11.3. Sewerage	□□□□□□
11.4. Garbage collection	□□□□□□
11.5. Home telephone	□□□□□□
11.6. Mobile telephone	□□□□□□
11.7. Internet	□□□□□□
11.8. Cable Television	□□□□□□
11.99.Other (specify)______________________	□□□□□□

0= Does not have
1= Deficient
2= Insufficient
3= Regular
4= Good
5= Excellent

12. The water received by the house comes from:
    12.1. Public mains ( )
    12.2. Water Well ( )
    12.3. River, stream, ditch or canal ( )
    12.4. Tanker ( )
    12.5. Rainwater ( )
    12.99.Other (specify)____

13. Has the incoming water been tested for quality?
    13.1. YES ( )
    13.2. NO ( )
    13.3. If your answer is YES, from which Agency or Institution__________
    ____

14. How is the house supplied with electricity?
    14.1. Public electric ( )
    14.2. Solar panel ( )
    14.3. Electricity generator (power plant) ( )
    14.99. Other (specify)____

15. What is the access route to the house?
    15.1. Cobblestone, paved or concrete street or roads ( )
    15.2. Cobbled street or road ( )
    15.3. Ballasted or ground road or street ( )
    15.4. Path, road, chaquiñán (shortcut) ( )
    15.5. River/sea/lake ( )
    15.99.Other (specify)____

16. What are the main furnishings in the house? Specify the number in each option

Property	Quantity	Property	Quantity
16.1. Wood stove		16.8. Television	
16.2. Gas stove		16.9 Living room furniture	
16.3. Induction stove		16.10. Dining room furniture	
16.4. Refrigerator		16.11. Laptop	
16.5. Washing machine		16.12. Computer	
16.6. Music equipment		16.13 Cellular	
16.7. Microwave			

16.99.Other (specify)____
____

17. How many families live in the house? [ ]

18. How many people per family live in this house?
    - 18.1. How many people belong to Family one ( )
    - 18.2. How many people belong to Family Two ( )
    - 18.3. How many people belong to Family Three ( )
    - 18.4. How many people belong to Family Four ( )
19. If there is more than one family, how is the food preparation distributed?
    - 19.1. Independent ( )
    - 19.2. All together ( )

ENVIRONMENTAL PROBLEMS

20. Do you think that there are enough green areas in your neighborhood?
    - 20.1. YES ( )
    - 20.2. NO ( )

21. If the answer is NO, what activities would you carry out to promote the conservation of green areas?
    - 21.1. Reforest ( )
    - 21.2. Plant ( )
    - 21.3. Maintenance of the green areas of the neighborhood ( )
    - 21.4. Gardening ( )
    - 21.5. Family gardens ( )
    - 21.99. Other (specify) ( )

22. Do you think there is environmental pollution in your neighborhood?
    - 22.1. YES ( )
    - 22.2. NO ( )

23. If the previous answer is YES, the existing contamination is in:
    - 23.1. River ( )
    - 23.2. Drinking wáter ( )
    - 23.3. Air ( )
    - 23.4. Floor ( )
    - 23.99.Other (specify)________________________( )

24. What are the factors that cause pollution in the river?
    - 24.1. For household waste ( )
    - 24.2. For industrial waste ( )
    - 24.3. For the breeding of animals ( )
    - 24.4. For pesticides on the surface of the river ( )
    - 24.5. Plastic waste ( )
    - 24.99.Others (Specify)________________________

25. What are the factors that cause contamination in drinking water?
    - 25.1. Poor water quality ( )
    - 25.2. Lack of hygiene in storage containers ( )
    - 25.3. Carelessness in the conservation of water (uncovered container ( )
    - 25.99.Other (Specify)_______________________________________

26. What are the factors that cause air pollution?
    - 26.1. Charcoal burning ( )
    - 26.2. Animals breeding ( )
    - 26.3. Oxidation lagoon ( )
    - 26.4. Garbage burning ( )
    - 26.5. Emission of toxic gases (companies) ( )
    - 26.6. Emission of toxic gases (vehicles) ( )
    - 26.7. Gaseous emissions from the landfill ( )
    - 26.99.Other (Specify)________________________
27. What are the factors that cause ground contamination?
    - 27.1. Heavy metals ( )
    - 27.2. Herbicides and pesticides ( )
    - 27.3. Chemical spills ( )
    - 27.4. Household waste (garbage) ( )
    - 27.5. Plastic waste ( )
    - 27.99.Other (Specify)________________________

28. Who do you think is responsible for the pollution?
    28.1. Local government GAD ( )
    28.2. Companies ( )
    28.3. Citizens ( )
29. During the winter season, your home has been affected by:
    29.1. Landslides ( )
    29.2. Flooding ( )
    29.3. None ( )
    29.99.Other (Specify)________________________

WORK TEAM DATA: (Required Information)

Name of surveyor:____Telephone:__________________

Career to which you belong:____

Name of the team supervisor:____

Career Coordinator:____

Date of data collection ____ ___

CODE OF THE SURVEYED HOUSE:____

______________________________ ______________________________
Supervisor Signature (before) Supervisor Signature (after)
